# Over-constrained kinematic of the medial compartment leads to lower clinical outcomes after total knee arthroplasty

**DOI:** 10.1007/s00167-020-06398-3

**Published:** 2021-01-02

**Authors:** Nicola Pizza, Stefano Di Paolo, Raffaele Zinno, Giulio Maria Marcheggiani Muccioli, Piero Agostinone, Domenico Alesi, Marco Bontempi, Stefano Zaffagnini, Laura Bragonzoni

**Affiliations:** 1 Clinica Ortopedica E Traumatologica II, IRCCS Istituto Ortopedico Rizzoli, Via Pupilli 1, 40136 Bologna, BO Italy; 2 Dipartimento Di Scienze Biomediche E Neuromotorie DIBINEM, Università Di Bologna, Via San Vitale, 40125 Bologna, BO Italy; 3 Laboratorio Di Biomeccanica, IRCCS Istituto Ortopedico Rizzoli, Via di Barbiano 1/10, 40136 Bologna, BO Italy; 4 QUVI, Università Di Bologna, Corso D’Augusto 237, 47921 Rimini, RN Italy

**Keywords:** Total knee replacement, Posterior stabilized, Kinematics, RSA, Clinical outcomes

## Abstract

**Purpose:**

To investigate if postoperative clinical outcomes correlate with specific kinematic patterns after total knee arthroplasty (TKA) surgery. The hypothesis was that the group of patients with higher clinical outcomes would have shown postoperative medial pivot kinematics, while the group of patients with lower clinical outcomes would have not.

**Methods:**

52 patients undergoing TKA surgery were prospectively evaluated at least a year of follow-up (13.5 ± 6.8 months) through clinical and functional Knee Society Score (KSS), and kinematically through dynamic radiostereometric analysis (RSA) during a sit-to-stand motor task. Patients received posterior-stabilized TKA design. Based on the result of the KSS, patients were divided into two groups: “KSS > 70 group”, patients with a good-to-excellent score (93.1 ± 6.8 points, *n* = 44); “KSS < 70 group”, patients with a fair-to-poor score (53.3 ± 18.3 points, *n* = 8). The anteroposterior (AP) low point (lowest femorotibial contact points) translation of medial and lateral femoral compartments was compared through Student’s *t* test (*p* < 0.05).

**Results:**

Low point AP translation of the medial compartment was significantly lower (*p* < 0.05) than the lateral one in both the KSS > 70 (6.1 mm ± 4.4 mm vs 10.7 mm ± 4.6 mm) and the KSS < 70 groups (2.7 mm ± 3.5 mm vs 11.0 mm ± 5.6 mm). Furthermore, the AP translation of the lateral femoral compartment was not significantly different (*p* > 0.05) between the two groups, while the AP translation of the medial femoral compartment was significantly higher for the KSS > 70 group (*p* = 0.0442).

**Conclusion:**

In the group of patients with a postoperative KSS < 70, the medial compartment translation was almost one-fourth of the lateral one. Surgeons should be aware that an over-constrained kinematic of the medial compartment might lead to lower clinical outcomes.

**Level of evidence:**

II.

## Introduction

A nonignorable percentage of patients is still not fully satisfied after total knee arthroplasty (TKA) surgery [10, 11, 22]. The aberrant kinematics of the operated joint has been claimed as a possible reason [4, 22]. Therefore, many new TKA designs have been introduced, aiming at recovering a more “physiological” knee motion [12, 13]. In particular, the “medial pivot” kinematics (i.e., larger anterior–posterior translation of the lateral femorotibial compartment with respect to the medial one) is associated with native knee joint motion [12]. Such a pattern has already been described in previous works either intraoperatively (through surgical navigation systems) or under weight-bearing conditions (through radiographic/fluoroscopic techniques) [2, 3, 16, 23]. Nevertheless, there is no substantial evidence of the association between this specific kinematic pattern and either better postoperative outcomes or patients’ satisfaction. So far, only two studies tried to cover this lack [2, 21]; moreover, only one of them focused on a cohort of patients with low postoperative outcomes [21], defined through statistical cluster analysis.

Hence, the purpose of the present study was to assess the postoperative clinical outcomes and femorotibial joint kinematics after TKA surgery. The hypothesis was that the group of patients with higher clinical outcomes would have shown postoperative medial pivot kinematics, while the group of patients with lower clinical outcomes would not.

The present study was the first that compared two groups directly defined through the Knee Society Score (KSS), one of the most used scores in clinical practice. Moreover, to authors’ knowledge, the cohort of patients investigated in the present study was the largest of the current literature.

The clinical relevance of the present study derives from the possibility to detect the specific kinematical patterns in the patients that reported poor clinical outcomes one year after TKA surgery. Such information could be of great help in identifying and reducing the number of unsatisfied patients.

## Materials and methods

### Patients selection

A cohort of 94 nonconsecutive patients who underwent TKA was enrolled and prospectively analyzed after providing informed consent. The patients were randomly selected from the waiting list for primary TKA of *Blinded for submission* Institute, through a random number generator, available on the web site of National Health System. The inclusion criteria were: (1) age (50–85 years old); (2) severe radiographic osteoarthritis (Kellgren-Lawrence grade 3 and grade 4); (3) patients scheduled for a primary TKA. The exclusion criteria were: (1) previous corrective osteotomy on the affected lower limb; (2) post-traumatic arthritis; (3) severe preoperative varus–valgus deformity (Hip knee ankle angle > 10°); body mass index > 40 kg/m^2^; (4) rheumatoid arthritis; (5) chronic inflammatory joint diseases; (6) patients with a prepathological abnormal gait (amputated, neuromuscular disorders, poliomyelitis, developmental dysplasia of the hip); (7) severe ankle osteoarthritis (Kellgren-Lawrence > 3); (8) severe hip osteoarthritis (Kellgren-Lawrence > 3); (9) previous total hip or ankle replacement; (10) unwillingness to take part in this study and providing Health Insurance Portability and Accountability Act (HIPAA) authorization; (11) incomplete clinical or kinematical assessment.

From the database of enrolled patients, those who had both kinematical and clinical assessments were selected. Furthermore, the patients with a medial-stabilized (MS) and a cruciate-retaining (CR) TKA implant were excluded (MS *n* = 25, CR *n* = 17). Overall, a cohort of 52 patients was included in the study. The mean age of the patients was 70.5 ± 7.4 years. The cohort included 29 right legs and 23 left legs, 28 females and 24 males, 48 varus and 4 valgus (average hip knee ankle: 5.4° ± 4.8°).

All patients were operated by the same surgeon with decades of experience in TKA surgeries with the same standard technique (medial parapatellar approach, mechanically adjusted alignment) [22] and a posterior-stabilized (PS) TKA with patellar resurfacing. The femoral component used was either a J-curve design (*N* = 14, Persona®, Zimmer Inc, Warsaw, IN, US) or gradually reducing radius (*N* = 38, Attune ™ Knee System, Depuy Synthes, Johnson and Johnson, Warsaw, IN, US).

### Kinematical and clinical assessments

**Fig. 1:**
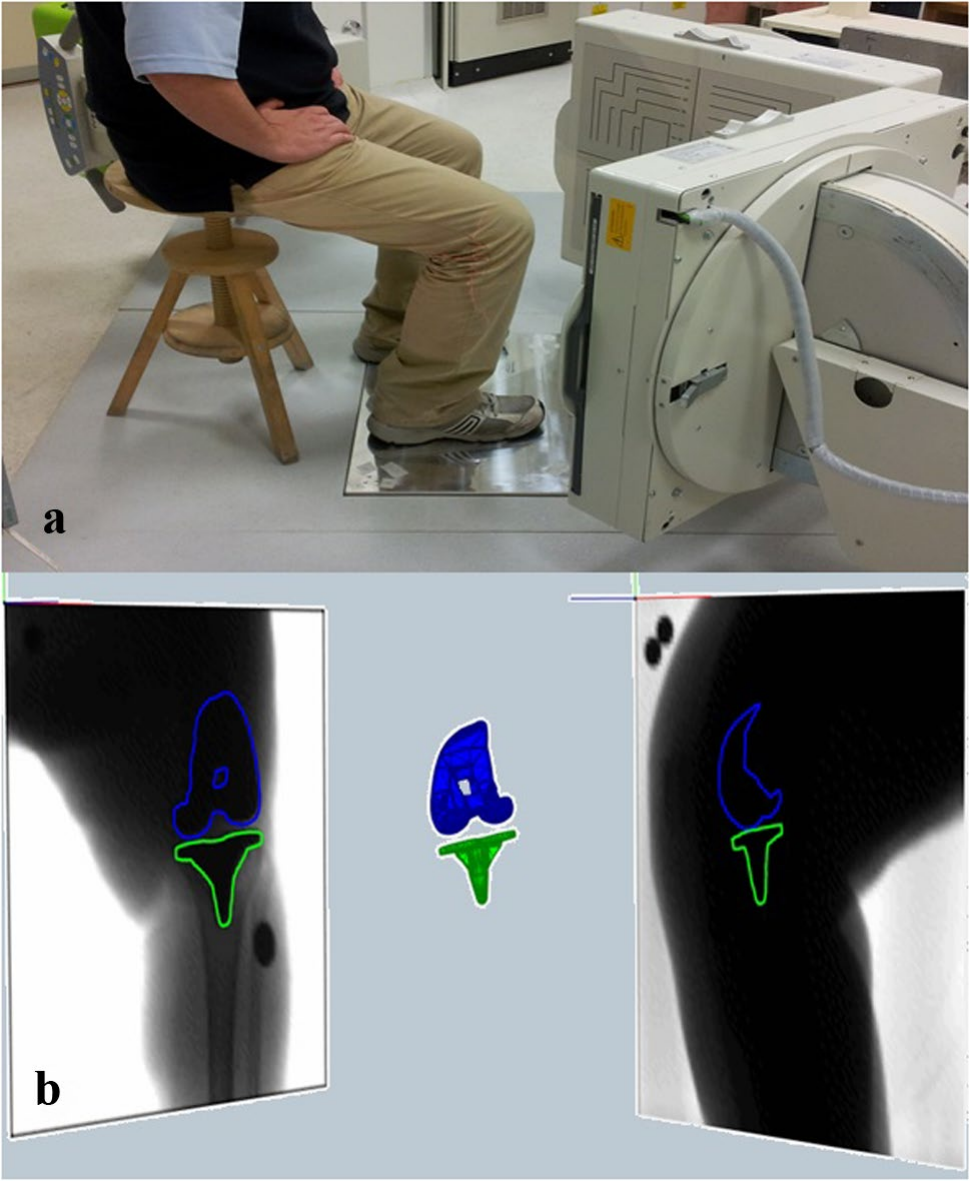
Setup of dynamic radiostereometric analysis (RSA). **a** RSA setup for the sit-to-stand motor task. **b** 3D environment of the RSA software for data processing, with contours segmentation on the radiographic images and semi-automatic model positioning

Patients were evaluated after a minimum of 9-month follow-up by model-based dynamic radiostereometric analysis (RSA). The RSA setup was analogous to those already published in previous articles from the same study group [7–9, 16]. Two radiographic tubes and two digital panels were positioned perpendicularly with respect to each other and synchronized to acquire two contemporary sets of images (Fig. 1a). The images were then postprocessed through a dedicated software by a single operator (*Blinded for submission*), where contours segmentation and CAD model positioning of tibial and femoral components were performed (Fig. 1b). The measurement accuracy of the validated dynamic RSA software is sub-millimetric (0.22 ± 0.46 mm and 0.26° ± 0.2° for the model position and orientation, respectively, according to the ISO-5725 regulation [14]), as evaluated in previous studies [1, 2, 6]. The operator’s repeatability (test–retest reliability) was evaluated through repeated tests under different image noise conditions [14]. The average error [5] was lower than 0.48 mm (95% CI 0.15–0.80 mm) for all the conditions.

All patients performed a sit-to-stand motor task, representing one of the most frequent daily activities: from the sitting position, the patient stood up without support from the upper limbs. The chair was made of a radiolucent material and was 40 cm high. The chair rising movement was not standardized to better simulate the natural movement that the patient would do in daily life.

The anteroposterior (AP) translation of the lowest medial and lateral femorotibial contact points, namely “Low Point kinematics”, was assessed for each patient’s movement through the dynamic RSA.

At the follow-up visit, the patients were clinically evaluated by the same senior orthopedic surgeon (*Blinded for submission*), who calculated the Knee Society Score (KSS). The KSS consists of a validated score, ranging from 0 to 100, and is considered good over 69 [18, 20]. The latter surgeon was not involved in the surgery and was not aware of the purpose of the study.

#### Statistical analysis

Based on the result of the KSS, patients were divided into two groups: in the “KSS > 70 group”, the patients with a good-to-excellent score; in the “KSS < 70 group”, the patients with a fair-to-poor score.

The low point kinematics evaluated through dynamic RSA was used to assess the presence of a medial pivot pattern (i.e., significantly higher AP translation of the lateral compartment as compared to the medial one) in the two groups. The medial and lateral AP translations were also separately compared between the two groups in order to assess differences in the range of motion.

The normal distribution of the data was verified by the Shapiro–Wilk test. Normally distributed data were expressed as mean and standard deviation, while categorical data were expressed as a percentage over the total. Differences between groups were expressed as absolute values, with 95% confidence intervals (CI). The Student’s *t* test was used to assess the statistical differences between the groups. The statistical effect of the following confounding variables on the KSS was also verified: sex, age, operated limb, prosthetic design. Differences were considered statistically significant for *p* < 0.05. Statistical analysis was performed in MATLAB (v2020a, The MathWorks Inc., Natick, Massachusetts, US).

An a priori power analysis was performed in G*Power (v3.1, Brunsbüttel, Germany) to calculate the sample size. Based on a previously published study with a similar aim [2], a mean difference of 4.2 mm between medial and lateral compartment AP translation with a standard deviation of 3.0 mm was considered. In order to reach a power value of 0.8 with *p* < 0.05, a minimum number of 10 patients per group was required.

### Ethics

All the patients involved in this research study signed informed consent forms. This study obtained the approval from Institutional Review Board (IRB) of Rizzoli Orthopaedic Institute (ID: 003,603 February 16th, 2016—Clinical Trial Gov ID: NCT02323386).

## Results

The average follow-up time for the RSA and clinical evaluation was 13.5 ± 6.8 months. All patients were able to perform the sit-to-stand task. Overall, 44 patients obtained a KSS of at least 70, considered good-to-excellent. For this group (KSS > 70), the average KSS was 93.1 ± 6.8 (Range 70–100). On the other hand, 8 patients obtained a KSS lower than 70, considered fair-to-poor. For this group (KSS < 70), the average KSS was 53.3 ± 18.3 (Range 30–69). The effect of sex, limb, and age was found to be not statistically significant (*p* > 0.05).

**Fig. 2:**
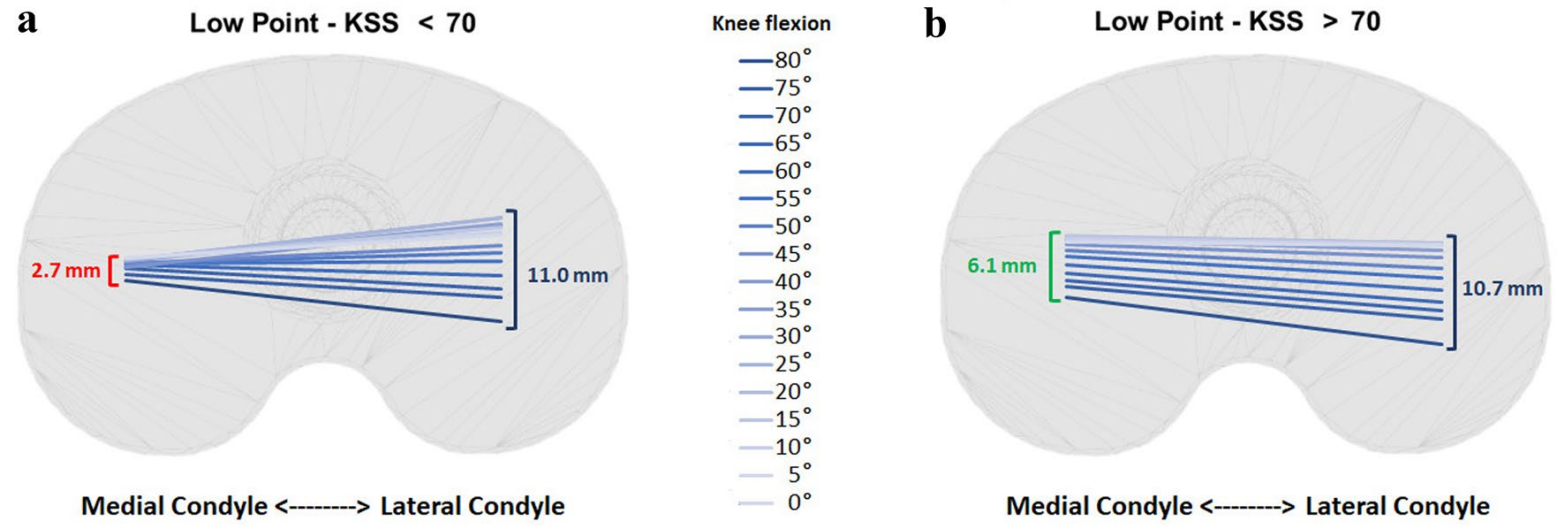
Low point kinematics according to Knee Score Society (KSS). Low point AP translation (in mm) of medial and lateral femoral compartment evaluated through dynamic radiostereometric analysis (RSA) during the sit-to-stand motor task in patients with **a** KSS < 70 and **b** KSS > 70

Regarding the low point kinematics, both groups showed the presence of a statistically significant (*p* < 0.05) medial pivot pattern (Table 1; Fig. 2). Furthermore, the AP translation of the lateral femoral compartment was not significantly different (*p* > 0.05) between the KSS > 70 and the KSS < 70 groups, while the AP translation of the medial femoral compartment was significantly higher for the KSS > 70 group (*p* = 0.0442) (Table 2; Fig. 2).

**Table 1:** Anteroposterior (AP) low point translation based on clinical outcome (Knee Society Score, KSS) evaluation

	Low point kinematics based on knee society score					
	KSS > 70 (*n* = 44)			KSS < 70 (*n* = 8)		
	Lateral compartment	Medial compartment	*p* value	Lateral compartment	Medial compartment	*p *value
Low point AP translation (mm)	10.7 ± 4.6	6.1 ± 4.4	< 0.0001*	11.0 ± 5.6	2.7 ± 3.5	< 0.0001*

*Represent statistically significant differences (*p* < 0.05)

**Table 2:** Difference in low point AP translation between KSS > 70 and KSS < 70 groups

Difference in low point ap translation between KSS > 70 and KSS < 70		
Compartment AP translation (mm)	Diff [95% CI]	*p* value
Lateral	− 0.3 [− 3.4–4.0]	ns
Medial	3.4 [0.1–6.7]	0.0442*

*ns* Means nonsignificant differences

*Represent statistically significant differences (*p* < 0.05)

A specific post hoc power analysis was performed to ensure the statistical effectiveness of the differences found between medial femoral compartment AP translation of the two groups. The power resulted in 0.89.

## Discussion

The most important finding of the present study was that the group of patients who reported a postoperative KSS < 70 showed a significantly lower AP translation of the low point of the medial compartment with respect to the group of patients with a KSS > 70. The hypothesis was partially confirmed since both groups of patients showed a “medial pivot” kinematics. Remarkably, in the group of patients with the KSS > 70, the AP translation of the medial compartment was more than half of the lateral one, while in the group of patients with KSS < 70 the medial compartment translation was one-fourth of the lateral one, on average (Fig. 2; Table 1).

In the present study, for the first time, clinical and functional outcomes after TKA were associated in a large cohort of patients to a kinematical analysis conducted in vivo, under weight-bearing conditions, during a daily life motor task.

Previous studies associating kinematics and clinical analyses were mainly performed intraoperatively [17, 23]. Warth et al. affirmed that the clinical success after TKA was not mandatorily related to the “medial pivot” pattern. However, it must be remembered that the kinematics was acquired intraoperatively, thus not necessarily mimicking the daily-life kinematics.

On the one hand, the patients of the present study reporting a good-to-excellent postoperative KSS showed medial pivot kinematics with a translation of the medial compartment that was almost half of the lateral one. This aspect means that the medial compartment was not constrained during the extension movement and could adjust its position following the lateral one without causing a paradoxical motion. On the other hand, the patients reporting a fair-to-poor KSS showed a medial compartment translation of 2.7 mm. Since the inlay incorporated in all patients’ TKA was not designed to stabilize the medial compartment, the latter resulted too constrained, even though this group showed medial pivot kinematics. From the authors’ perspective, this could be a reason for the lower clinical results. Previous clinical studies also suggested that TKA designs with symmetric inlays on medial and lateral compartment reported lower postoperative outcomes in the presence of an over-constrained medial compartment [15, 22].

Only two previous studies associated clinical outcomes and closed kinetic chain movement kinematics using a fluoroscopic or radiographic methodology [2, 21]. Apparently, both studies seem to contradict the finding of the present study. Alesi et al. [2] found a negative correlation between clinical outcomes and the medial compartment translation in the same motor task. However, this result was obtained by analyzing a MS TKA, i.e., a substantially different TKA design. Indeed, MS TKAs have a high congruent medial compartment inlay to stabilize the medial condyle and a flat lateral one to allow higher translation. Van Onsem et al. [21] affirmed that patients with low scores experienced more anterior translation on the medial side during mid-flexion and less posterior translation on the lateral side during deep flexion as compared to patients with high scores, thus resulting in a “lateral pivot” pattern [3]. As in the present study, lower clinical outcomes were found in the presence of boundary kinematical patterns.

In the present study, it was possible to identify specific kinematical patterns objectively related to patients with low outcomes. These results were obtained on a cohort of 52 patients; thus, nearly double of current literature [2, 3, 21]. Such an investigation might crucially contribute to accounting for the nonignorable percentage of dissatisfied patients after TKA. Unfortunately, in the present study, it was not possible to detect a specific cut off of medial compartment translation, which classified patients into the two groups. Further analyses are needed to detect a specific and reliable cut off that might predict patients’ satisfaction and potentially correlate it to the data from daily clinical practice diagnostic devices.

Some limitations are present. First, the postoperative kinematical data were collected only for one movement repetition per patient. This was done for ethical reasons in order to reduce patients’ X-ray exposure as much as possible. However, it must be remembered that patients were asked to perform the task three times before the data acquisition to get confidence with the dynamic RSA setup. Second, the population included in the study received a single TKA design, the PS design. This was done to reduce the confounding effect given by the influence of TKA design on knee kinematics. Thus, no conclusions can be drawn for other specific TKA designs. However, differently from previous studies where PS and CR designs were pooled together [19, 21, 23], the investigated cohort of the present study was highly homogenous. This might be considered a strength rather than an issue. Third, the sample size could be considered small for a clinical investigation. However, as already underlined, the cohort of patients under investigation was larger (nearly double) than the one of previously published studies with the same ratio [2, 19, 21]. Moreover, a power analysis ensured the effectiveness of the statistical findings for the number of patients involved.

The clinical relevance of the present study is that medial pivot kinematics is associated with good-to-excellent postoperative clinical outcomes as long as there is no over-constraint of the medial compartment. Further studies are needed to confirm the results shown in the present study at longer follow ups and correlate the postoperative kinematics to intraoperative one to detect “dangerous” kinematical patterns and correct them intraoperatively.

## Conclusion

The group of patients with a postoperative KSS < 70 one year after TKA surgery showed a significantly lower AP translation of the low point of the medial compartment with respect to the group of patients with a KSS > 70. During TKA procedure, surgeons should be aware that over-constrained kinematics of the medial compartment might lead to lower clinical outcomes and should avoid it by performing an adequate release of the medial structures if needed.

## Abbreviations

ACL: Anterior cruciate ligament
AP: Anteroposterior
CR: Cruciate retaining
KSS: Knee society score
MS: Medial stabilized
PS: Posterior stabilized
RSA: Roentgen stereophotogrammetric analysis
TKA: Total knee arthroplasty
